# Spatial Clustering of Tuberculosis Incidence in the North of Iran

**DOI:** 10.5539/gjhs.v6n6p288

**Published:** 2014-09-23

**Authors:** Jamshid Yazdani-Charati, Hasan Siamian, Anoushirvan Kazemnejad, Mohammad Vahedi

**Affiliations:** 1 Department of Biostatistics, Health Sciences Research Center, Faculty of Health, Mazandaran University of Medical Sciences, Sari, Iran; 2 Health Information Management Department, Health Sciences Research Center, School of Allied Medical Sciences, Mazandaran University of Medical Sciences, Mazandaran, Sari, Iran; 3 Tarbiat Modares University, Tehran, Iran; 4 Department of Microbiology, School of Medicine, Mazandaran University of Medical Sciences, Sari, Iran

**Keywords:** tuberculosis, incidence rate, GIS Soft ware, core area, Standard Morbidity Ratio (SMR)

## Abstract

**Background and Purpose:**

Tuberculosis (TB) poses a serious threat to public health throughout the world but disproportionately afflicts low-income nations. The aim of this study is to identify the high-risk areas in Mazandaran province (North of Iran) in helping the heath programmer for the best intervention.

**Materials and Methods:**

This is an ecological study conducted from 1999 through 2008. The sample included 2444 Tuberculosis (TB) patients. The variables were age, gender, type of disease and residential location, analyzed by descriptive statistical methods and spatial analysis to identify cluster of disease incidence. Geographical information system software applied to map of smooth rate of TB.

**Results:**

Of 2444 registered patients, 1283 (52.5%) were male. The data showed 61% urban and 96.4% of them with the Iranian nationality. There was insignificant difference between genders, but the main difference was observed between locations that are the incidence rate in the Tonekabon and Behshahr cities were 30% higher than mean incidence rate of Mazandaran province (P-value<0.05). The comprising chance of acquiring infection between urban and rural was 1.46 with confidence interval of 95% (1.35, 1.59).

**Conclusion:**

Geostatistical method showed spatial variability of TB incidence rate in all districts and identifying high-risk area (core areas). The most important core of TB incidence has been noticed in the eastern boundary of Mazandaran in the city of Behshahr which is due to proximity to Golestan Province. The incidence rate of TB in Behshahr city is about two times more than the number observed in Mazandaran province. Lower TB incidence rate has been observed in Golestan province is because there is usually a delay in the diagnosis of the disease especially in the positive smear patients.

## 1. Introduction

TB remains one of the most dangerous infectious diseases with more than 9 million cases annually and with more than 1.7 million mortalities per year (Cerf, 1994; Hannu, GianLuca, & Olesen, n.d.). In 2012, 8.6 million people fell ill with TB and 1.3 million of them died. Over 95% of TB deaths occur in low- and middle-income countries, and it is among the top three causes of death for women aged 15 to 44 (WHO, 2014).

A Spatial cluster might be defined as an overload of events or of values in geographic area (Li et al., 2011). Spatial cluster analysis plays an important role in quantifying geographic variation patterns. It is commonly used in disease surveillance, spatial epidemiology, population genetics, landscape ecology, crime analysis and many other fields, but the underlying principles are the same (Jacquez, 2008).

Tuberculosis (TB) poses a serious threat to public health throughout the world but disproportionately afflicts low-income nations. Approximately 90% of TB cases among adults can be attributed to reactivation TB (Sia & Wieland, 2011). Rapid expansion of the standardized approach to tuberculosis diagnosis and treatment that is recommended by WHO allowed more than 36 million people to be cured between 1995 and 2008, averting up to 6 million deaths. Yet tuberculosis remains a severe global public health threat. There are more than 9 million new cases every year worldwide, and the incidence rate is falling at less than 1% per year. Although the overall target related to the Millennium Development Goals of halting and beginning to reverse the epidemic might have already been reached in 2004, the more important long-term elimination target set for 2050 will not be met with present strategies and instruments. Several key challenges persist. Many vulnerable people do not have access to affordable services of sufficient quality. Technologies for diagnosis, treatment, and prevention are old and inadequate. Multidrug-resistant tuberculosis is a serious threat in many settings (Lönnroth et al., 2010). The main treatment for TB is medical. More than 90% of TB cases can be treated by new chemotherapeutics. However, medical treatment alone fails in 20-40% of the MDR-TB patients (Mohsen, Abou Zeid, & Haj-Yahia, 2007; B. Xie, Yang, He, D. Xie, & Jiang, 2013). When WHO declared tuberculosis a global emergency in 1993, the resulting attention helped to mobilize new private and public resources for tuberculosis research. Tuberculosis today is humanity’s greatest killer, and it is out of control in many parts of the world. The disease, preventable and treatable, has been grossly neglected and no country is immune to it (“WHO calls tuberculosis a global emergency. Los Angeles Times (LA), April 24, 1993. http://articles.latimes.com/1993-04-24/news/mn-26683_ 1_global-emergency “, 1993)

The last 10 years, there has been a refreshed international interest for research in TB, and this has resulted in the launch of several new initiatives by national and international organizations, private charities and pharmaceutical companies. Then World Health Organization (WHO) has stated a global plan to stop TB: 2006–2015” which was certified in 2005 by the Stop TB Partnership (Dyson, 2004). Identification of geographical areas with on-going disease transmission, using GIS and spatiotemporal statistical analyses, have become indispensable (Carla Nunes, 2007).

Spatial clustering methods are concerned with the identification of greater density of occurrences of a phenomenon in certain places. These methods have intensively applied in several areas such as demography, toxicology, criminology, etc.

Disease clustering is a method of main attention to epidemiologists that have studied for many decades. For an effective disease management it is essential to know when, where and to what degree a disease is present. During the last decade, there has been a huge and fast development of spatiotemporal clustering and it applied to health such as: estimation of infectious diseases, cancer, rheumatisms, diabetes and accidents among others. Conjugation between classical approaches of space-time clusters and geostatistical methodologies is relatively application of spatially correlated data. Berke (2004) estimated spatial risk functions from regional count data (Berke 2004). Goovaerts and Jacquez (2005) presented neutral models (based on Sequential Gaussian Simulation) for the detection of changes in mortality rates across space and time used the local Moran I Statistics (Goovaerts & Jacquez, 2005). Goovaerts (2006) used Poisson kriging and p-field simulation to assess cancer mortality risk (Goovaerts, 2006). In the present study, the objective is to map spatial cluster of TB incidence in Mazandaran (Norther Iran) in order to determine when and where unusually high concentrations of new cases occurred, considering the gender distributions in local populations from 1999 through 2008. Data Structure, Mazandaran (North of Iran), bordering the Caspian Sea on the north (Encyclopedia Britannica, 2014). Mazandaran is one of the thirty provinces of Iran and is located in north of Iran. The east and west neighbors are Golestan and Guilan provinces, respectively. Mazandaran (provinces, Iran) has 16 township and 44 districts. We used tuberculosis data, from tuberculosis registry, and then data entry has been done. In this study, diagnosis tests for TB were TB skin test, chest X-ray and sputum test. In all, 2444 patients who have been registered in all health centers of this province, were 2444 persons recorded separately, based on sex, age, gender, ethnicity, living place (rural or urban) and type of TB disease, then, the incidence rate of each district was determined.

## 2. Methods

In this study descriptive epidemiological methods were used to summarize TB incidence data. For calculating people at risk, censuses from 1996 through 2006 of Mazandaran have been applied. Technically, this usage corresponds to an incidence proportion rather than a rate, but is very common because this incidence proportion is a population-based estimate of (average) individual risk within the study population. Most diseases affect people of certain ages disproportionately, therefore rate of standardization is a mechanism to adjust summary rates to remove the effect of known risk factors and make rates from different populations comparable. Then the standardized morbidity ratio (SMR) calculated based on age and gender.

We want to predict SMR of TB for each district from either data at individual locations or data associated with the regions themselves. One of the most important aims of the research was identifying spatial cluster of TB incidence in Mazandaran province. The most important index of spatial cluster analysis is determination of spatial autocorrelation to summarize the degree to which similar observations tend to occur near to each other. Identifying spatial pattern of TB incidence and exploration of hot and cold spot locations in this paper was done by calculating Moran’s I. smoothing methods helps researcher to predict a reliable estimate of incidence rate specifically for unreported location. If denotes similarity between data values, Y_i_ and Y_j_, and let denote a weight describing the proximity between locations *i* and *j*, for *i* and *j* = 1,…,N.

In general the extent of similarity is represented by the weighted average of similarity between areal units: Global indexes of spatial autocorrelation are built on this basic form

We often have the weight of as a spatial proximity matrix; in our research are binary connectivity matrixes, whose elements are Geographic information systems (GIS) applied not only by disease mapping but also by spatial analyses, such as spatial clustering and cluster detection (Berke, 2004; Nunes, 2007; Stop TB Partnership, WHO, 2006; Goovaerts & Jacquez, 2005). In this context, clusters defined as unusual concentrations of health events in space. We selected grid equal to 500 meters (Spherical Semivariogram Model). A special application of infectious disease mapping is the analysis of core areas (the geographical analogues of areas in which incidence rate and transmission are unusually high). GIS can be used in identifying core areas and analyzing the patterns of disease transmission within populations (Thomas & Tucker, 1996).

## 3. Results

In this survey considering the epidemiological variables such as age, gender, residence and race, 2444 patients were identified, the 1283 males (52.5%), 1161 females (47.5%), 61 % of from urban areas, 39% from rural areas, 96.4% of them from Iran and the rest of them from Afghanistan. The mean age of male TB patients was 47.5±20 year and in female 46.3±16.21 year. Generally about 1562 (73%) of the patients were in age range of 15-64 yrs and 597 (24%) patients were above 64 years old and 79 (3%) patients lower than 15 yr. Table 1 shows the incidence rate of TB in Mazandaran province was 10.69 per 100000 persons (rates of disease in the throughout this paper are in 100000 persons). The maximum incidence rate of 19.39 belongs to Behshahr (a city in the east of Mazandaran province) and the minimum incidence rate of 6.45 attributed to Jooybar (a city in the center of Mazandaran province).

**Table 1:** All Type of TB incidence Rate in cities of Mazandaran Province during 1999-2008 among 100000 people

County Name	extra pulmonary TB rate	pulmonary smear Negative TB rate	pulmonary smear positive TB rate	All type of TB rate
Amol	2.07	2.99	4.49	9.55
Babol	2.66	3.94	3.99	10.59
Babolsar	2.38	1.37	3.70	7.45
Behshahr	5.51	4.12	9.76	19.39
Chalous	2.41	1.64	4.56	8.61
Galogah	0.52	0.26	10.70	11.48
Ghaemshahr	2.35	2.77	5.29	10.41
Jooybar	2.20	1.03	3.22	6.45
Mahmud Abad	1.37	1.37	7.31	10.06
Neka	2.34	3.46	7.01	12.81
Noor	1.96	1.18	4.12	7.27
Noshahr	2.30	1.95	3.89	8.14
Ramsar	3.35	1.98	5.63	10.95
Sari	4.32	3.06	5.03	12.41
Savadkuh	1.86	2.32	4.65	8.83
Tonekabon	2.45	3.94	4.20	10.59
Province	2.81	2.84	5.04	10.69

The highest incidence rate of pulmonary smear positive TB was accounted in Galogah and the lowest rate in Jooybar with 10.7% and 3.22%respectively. The highest and lowest incidence rates of extra pulmonary TB were as follows in Behshahr and Galogah with the rate of 5.51 and 0.52 respectively. The maximum rate of negative smear pulmonary TB in Jooybar was 4.12, and the minimum Galogah with the rate of 0.26. We identified 53% of our patients were clustered.

**Figure 1:**
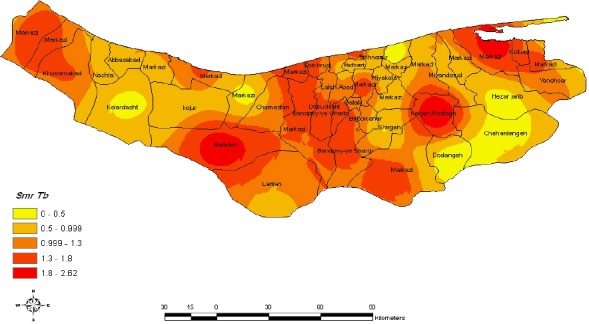
Geographic distribution of age adjusted tuberculosis Morbidity rates the fill color in each district represents the age and sex adjusted morbidity rate per 100000 person years recorded over the period 1999-2008

After using GIS soft ware and applying interpolation method in district level to improve precision, the cores of TB infection identified. Eight high risk cores were identified in Mazandaran province, that is, Baladeh district of (at the Noor county), central district of Ramsar and Tonekabon, center district of Babol, Kolijan Rastagh district of Sari and central district of Behshahr with 1.8 -2.8 times higher than the total incidence rate of the province that accustomed due to age and gender, and presented here with SMR (Figure 1). In addition, during this period, there were insignificant changes between different years.

## 4. Discussion and Conclusions

Like the other countries, health ministry of Iran performed WHO recommended DOTS strategy (directly observed treatment, short-course) to control TB. To prevent TB, Bacillus Chalmette-Guerin (BCG) vaccine has widely used for all children in initial days after birth. But it offers limited protection to adults and its overall efficacy is considered modest (Colditz, Brower, Berkey et al., 1994) Identifying high-risk location is a good approach for health programmer for appropriate intervention. The reported incidence rate of TB in Mazandaran during 1999-2008 was 10.69 lower than the other places of Iran indicating 13.4. in 2008, these rates in the neighboring provinces Golestan and Guilan were 44.8 and 15.7 respectively. Among all the provinces, after Sistan and Baluchistan, Golestan had the highest incidence rate (“General Report of Tuberculosis, Health Ministry of Iran.,” 2009). Considering the age of patients in this surveys, the mean age of the patients was higher than 42 years reported in Ardebil (a city in the north west of Iran) (Amani & Bashiri, 2007). These results showed that the health service in Mazandaran was better than Ardebil or may be the identifying rate of TB cases less than from them. But there were no significant differences between mean age of Mazandaran and Golestan and Guilan provinces. In addition a person infected TB but who stay healthy and never become sick. So if a person’s immune system is affected (e.g. through HIV infection, chemotherapy for cancer, old age, stress, etc.). Since 73% of the patients were the range of 15-64 years, this condition decreased the income of the province because they could not work for 3 to 4 months. The highest rate of death due to TB occurred in this age range, therefore about 80% of economical loss was sustained to this age group.

These results are in agreement with that of other studies and no significant differences were witnessed between the genders. The result of this study is different from that of other relevant studies performed in the neighboring provinces. In a study the amount of incidence rate of TB in female was higher than male (Baker et al., 2008; Jaine et al., 2011). The disease was more incidental among the urban dwellers. The result of this study is similar to the study performed in Ardebil, and different from that of Birjand city it could be due to two factors: appropriate weather condition and proper accomplishment of DOTS methods in treating the patient. Table 1 and Figure 1 show the highest incidence rate of TB in Behshahr which indicates the geographical correlation of this disease. Because this city is proximal to Golestan province. The rate of positive smear pulmonary TB incidence is a main index of TB infection. In this regard, our finding (7.6%) was higher than the similar studies performed in the other regions of Iran (7.6). These indexes were reported 22.8 for Golestan, 7.7 for Guilan during 2006. Of course, this index in Behshahr, Galogah and Neka were the highest measure of them in Mazandaran due to proximity to Golestan Province. Identifying core areas are a good approach to help heath programmer for appropriate interventions. The most important core of TB incidence in the east boundary of Mazandaran was Behshahr city due to proximity of Golestan the incidence rate of TB in this city about two times than that of Mazandaran and lower TB rate infection may be due to delay in diagnosis of TB in Mazandaran townships by use the other core of TB diseases was Kolijan Rastagh in Sari, Baladeh of Noor, and Center Babol owing to presence of industrial units near these districts. Tuberculosis (TB) is generally considered to be linked to industrialisation and urbanisation (Tremblay, 2007). Another cause of core in these districts, are migration of young people. The elderly population have high chance for TB infection (Rajagopalan, 2001), due to different immune system, and other diseases (Perez, Sheton, & Restepto, 2006; Roland et al., 2007) and Neck cancer cases (Kim et al., 2005). Another factor associated with TB infection is silica dust exposure. It leads to silicosis that is associated with TB infection. In The Center districts of Tonekabon, Ramsar, Noshahr, Mahmud Abad and Sorkhrood, core TB incidence have been seen because of overcrowding (Bakeret al., 2008), being tourism area. Identifying core of TB incidence is good instruction for health programmer to select the best intervention and estimate lower risk for allocating resources. In the population under our study, the diagnostic test for HIV has not been done. Despite, TB infection is one of the most important causes of death among HIV patients. Health ministry of Iran tried to follow WHO stop TB program. Nevertheless, case detection is lower than 65%. One reason is that health care employees do not have appropriate knowledge about TB.

Using spatial analysis and applying GIS soft ware for disease mapping can be an effective method for identifying high-risk areas that could be an appropriate method intervention for health programmer.
